# Severe Recurrent Hemorrhage of an Oncogenic Vertebrobasilar Junction Aneurysm Following Stent-Assisted Coil Embolization in Metastatic Testicular Choriocarcinoma

**DOI:** 10.7759/cureus.114551

**Published:** 2026-08-14

**Authors:** Haksoo Lee, Yongwoo Sim, Junghyun Park

**Affiliations:** 1 Neurological Surgery, Kosin University Gospel Hospital, Busan, KOR

**Keywords:** leptomeningeal carcinomatosis, oncogenic cerebral aneurysm, stent-assisted coiling, testicular cancer, vertebrobasilar junction

## Abstract

Oncogenic cerebral aneurysms (OCAs) are rare, catastrophic lesions typically arising from distal middle cerebral artery (MCA) branches. We present a case involving an atypical OCA at the vertebrobasilar (VB) junction secondary to metastatic mixed testicular cancer predominantly comprising choriocarcinoma, which resulted in fatal rebleeding despite initial stent-assisted coil embolization. A male in his 30s with metastatic choriocarcinoma and leptomeningeal carcinomatosis presented with ambiguous subarachnoid hemorrhage (SAH) on initial MRI. Subsequent CT angiography (CTA) identified an outpouching suspicious for an aneurysm at the VB junction communicating with bilateral VAs, without localized acute SAH. Stent-assisted coil embolization was performed. On postoperative day six, the patient suffered unexpected catastrophic rebleeding. Despite rescue efforts, angiography revealed no intracranial flow, consistent with brain death. Neurointerventionists should be aware that OCAs can manifest in atypical locations like the VB junction. Due to the high risk inherent in their pathophysiology, patients with metastatic choriocarcinoma involving the brain should undergo proactive intracranial vasculature screening.

## Introduction

Oncogenic cerebral aneurysms (OCAs) are rare vascular complications associated with malignancy and are often associated with a poor prognosis, depending on the underlying tumor histology [1]. They are most commonly reported in patients with cardiac myxoma, choriocarcinoma, and other malignancies [1]. OCAs related to cardiac myxoma demonstrate a relatively favorable prognosis, whereas those with choriocarcinoma carry a substantially higher mortality rate [1]. Most OCAs arise in distal branches of the middle cerebral artery (MCA) or anterior communicating artery, sometimes hidden within cortical sulci [1]. They are typically characterized by small size, irregular margins, and fusiform dilatation [1,2]. Recently, rare malignancies such as renal cell carcinoma have also been associated with OCAs [3]. Owing to their atypical locations and morphologies, OCAs may be overlooked in clinical practice [1, 3]. Unlike conventional degenerative or infectious aneurysms, oncogenic aneurysms result from direct tumor cell invasion into the intima. In particular, metastatic choriocarcinoma possesses an exceptionally aggressive and angioinvasive nature, rapidly compromising the structural integrity of the arterial wall. We report a rare case of saccular OCA at the vertebrobasilar (VB) junction secondary to metastatic testicular cancer with leptomeningeal carcinomatosis, which resulted in unexpected fatal rebleeding despite initial treatment with stent-assisted coil embolization.

## Case presentation

**Figure 1:**
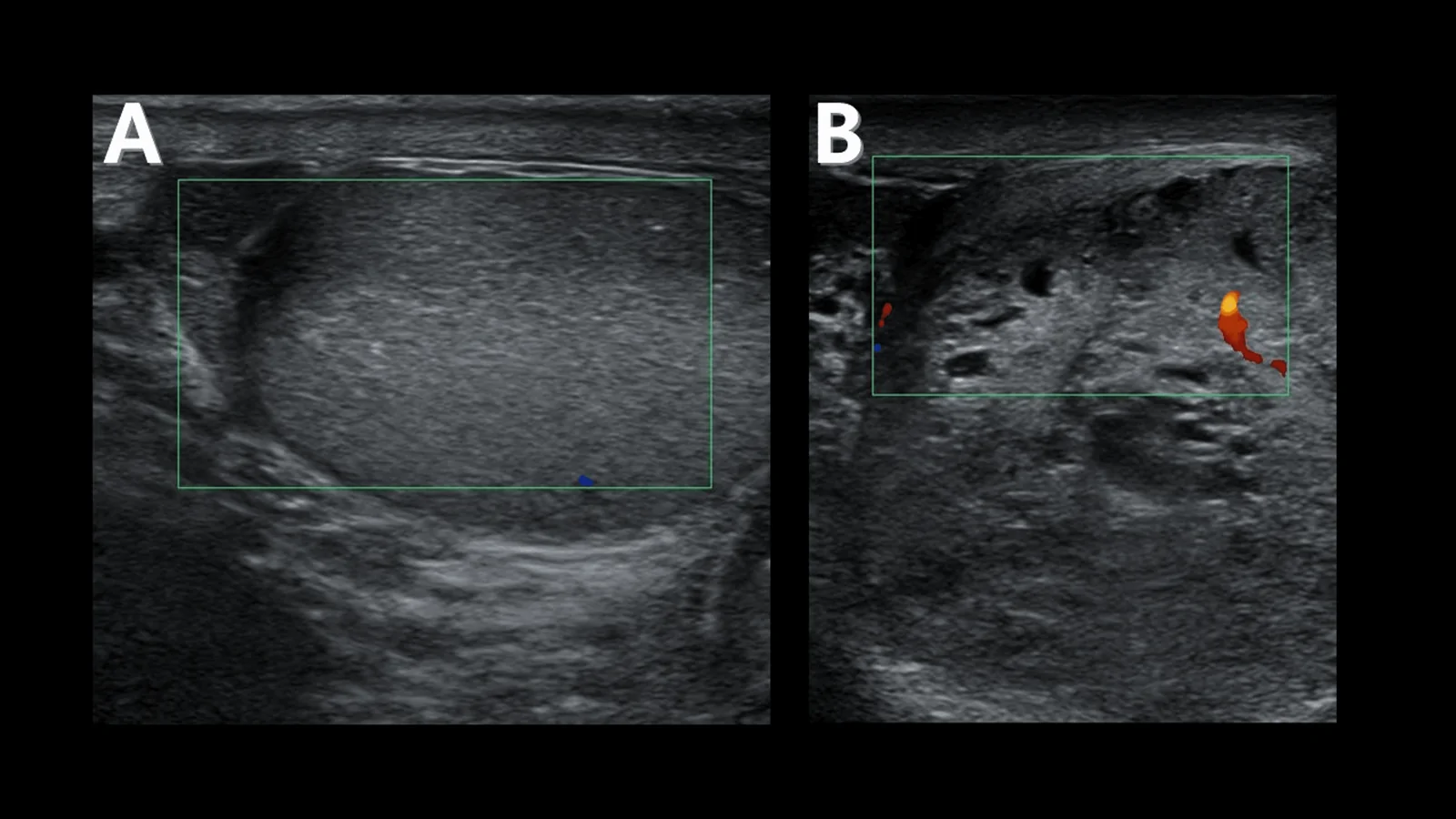
Color Doppler ultrasonography of the testis (A) Color Doppler sonography of the right testicle demonstrates a solid, heterogeneous intratesticular mass with minimal internal vascular flow, reflecting extensive central necrosis. (B) Additional imaging reveals multiple cystic spaces within the intratesticular mass, demonstrating a hypervascular pattern characteristic of viable tumor components

**Figure 2:**
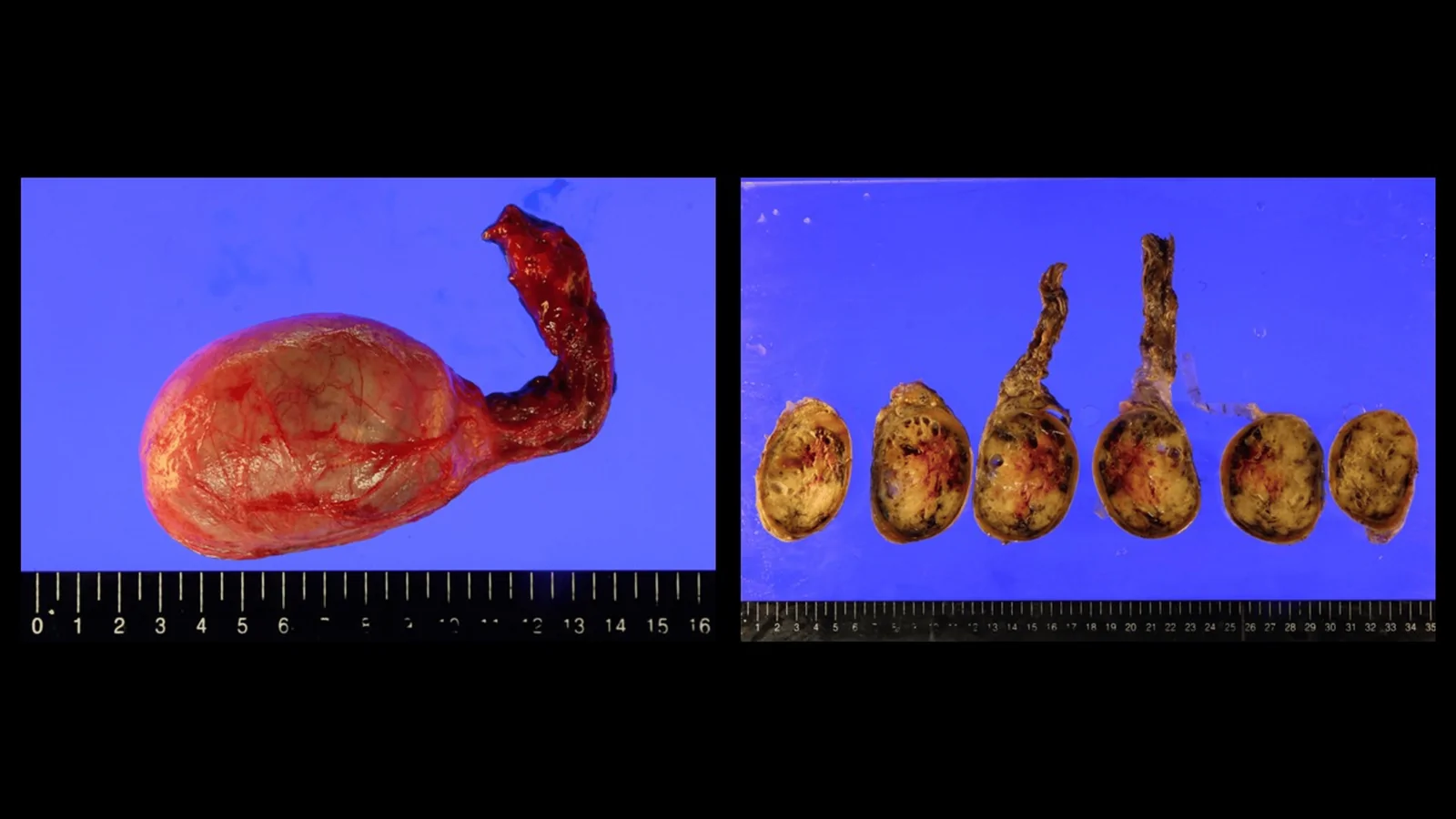
Gross pathological images of the testis following total orchiectomy Gross examination of the cross-sectioned testis reveals a markedly heterogeneous tumor mass. The lesion demonstrates a complex admixture of pale, fleshy solid areas and multiple cystic spaces, interspersed with extensive regions of hemorrhage and necrosis. Notably, the prominent widespread hemorrhagic and necrotic changes strongly indicate the presence of a choriocarcinoma

The patient was a male in his 30s with metastatic testicular cancer and leptomeningeal carcinomatosis who presented with headache (Glasgow Coma Scale (GCS) score: 14). He initially presented with testicular swelling, and ultrasonography revealed a large, heterogeneous mass suggestive of malignancy (Figure 1). Preoperative tumor markers were markedly elevated, with a serum β-hCG level of 17,791 mIU/mL and an alpha-fetoprotein level of 888 ng/mL, while lactate dehydrogenase (LDH) was within the normal range (182 U/L) (Table 1). The patient had undergone radical orchiectomy one year before the onset of subarachnoid hemorrhage (SAH). Histopathological examination confirmed a mixed germ cell tumor composed of yolk sac tumor, choriocarcinoma, and teratoma (Figure 2).

**Table 1:** Serial changes of tumor markers and laboratory findings during the clinical course This table summarizes the serial changes in tumor markers, notably highlighting a dramatic beta-HCG surge before the onset of SAH and elevated LDH levels. This profound beta-HCG surge indicates the aggressive activation of metastatic choriocarcinoma, while the LDH levels reflect ongoing tumor cell proliferation SAH: subarachnoid hemorrhage; hCG: human chorionic gonadotropin; AFP: alpha-fetoprotein; PSA: prostate-specific antigen; LDH: lactate dehydrogenase

Parameter	Initial presentation (day 1)	Intermediate follow-up	One month before SAH (peak surge)	Reference range (unit)
Beta-hCG	17,791.00	997.0-1,413.0	264,033.00	< 5.0 (mlU/mL)
AFP	888	2.19-2.60	-	< 8.0 (ng/mL)
PSA	0.67	-	-	< 4.0 (ng/mL)
LDH	182	684-1,263	-	140-280 (U/L)

**Figure 3:**
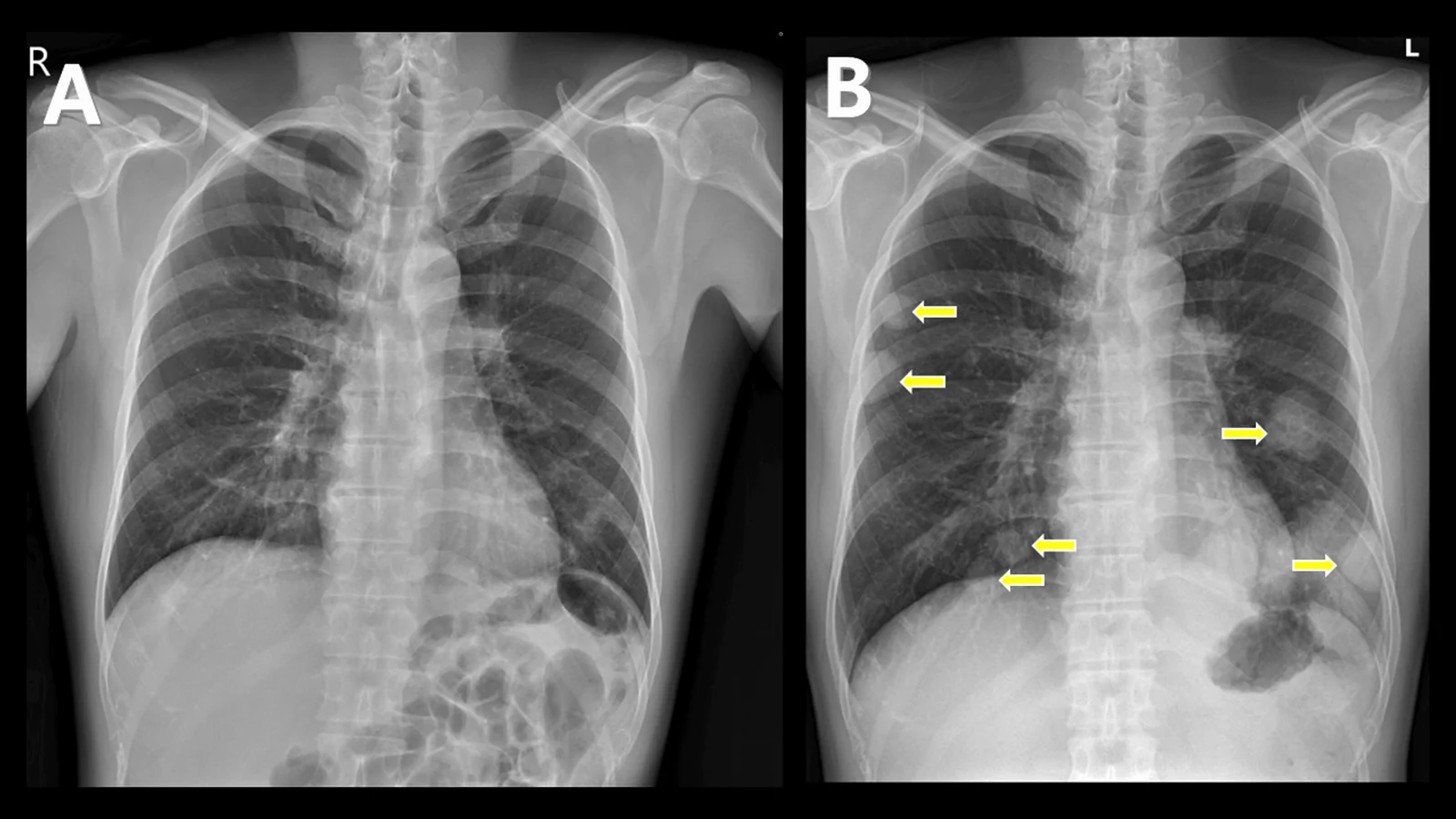
Initial and follow-up chest X-ray images (A) The previously performed chest X-ray shows no mass lesions. (B) The follow-up chest X-ray demonstrates multiple masses throughout both lung fields (arrows)

**Figure 4:**
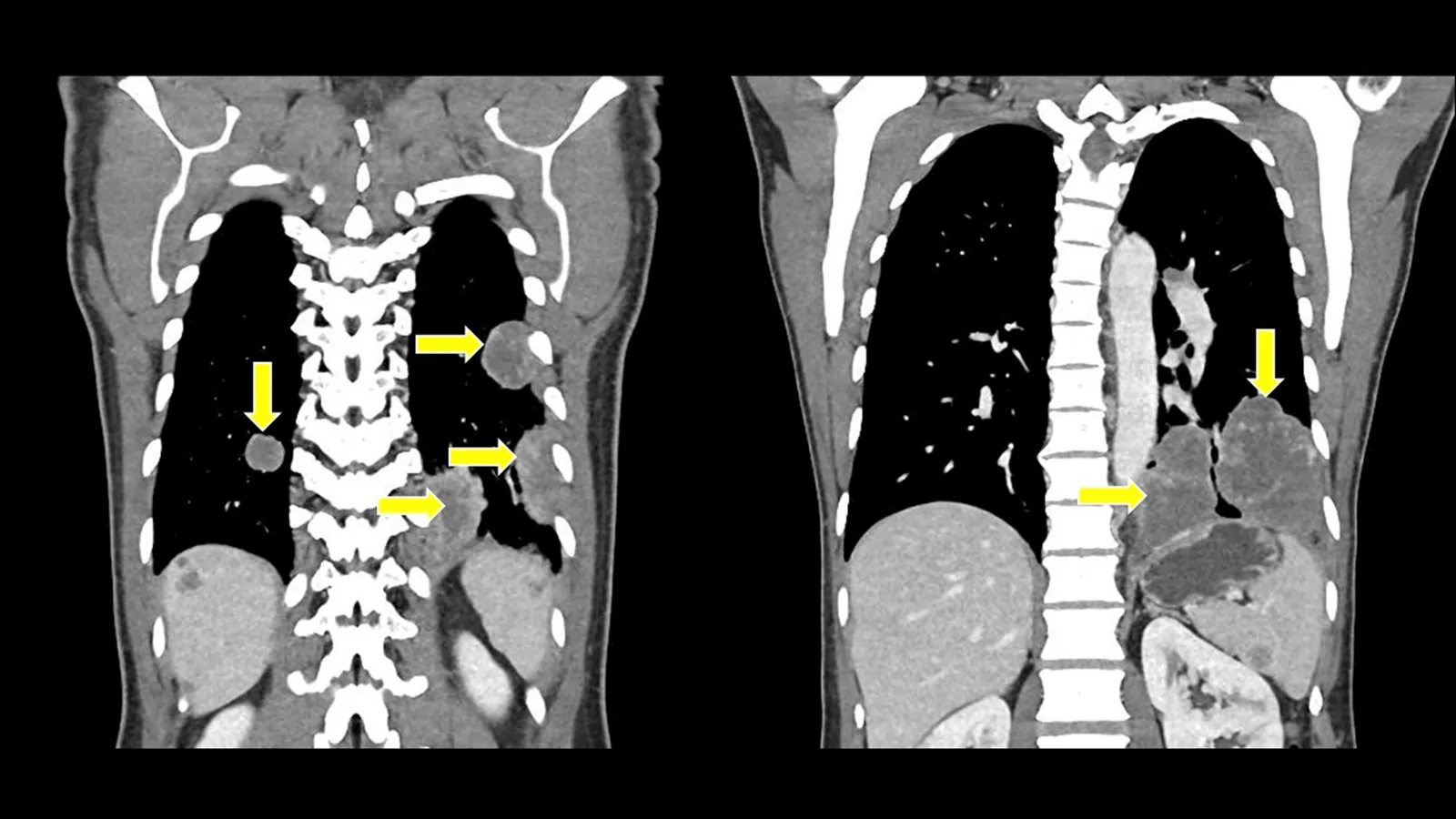
Contrast-enhanced CT of the chest Coronal contrast-enhanced CT images show multiple nodular masses, as seen on the chest X-ray CT: computed tomography

**Figure 5:**
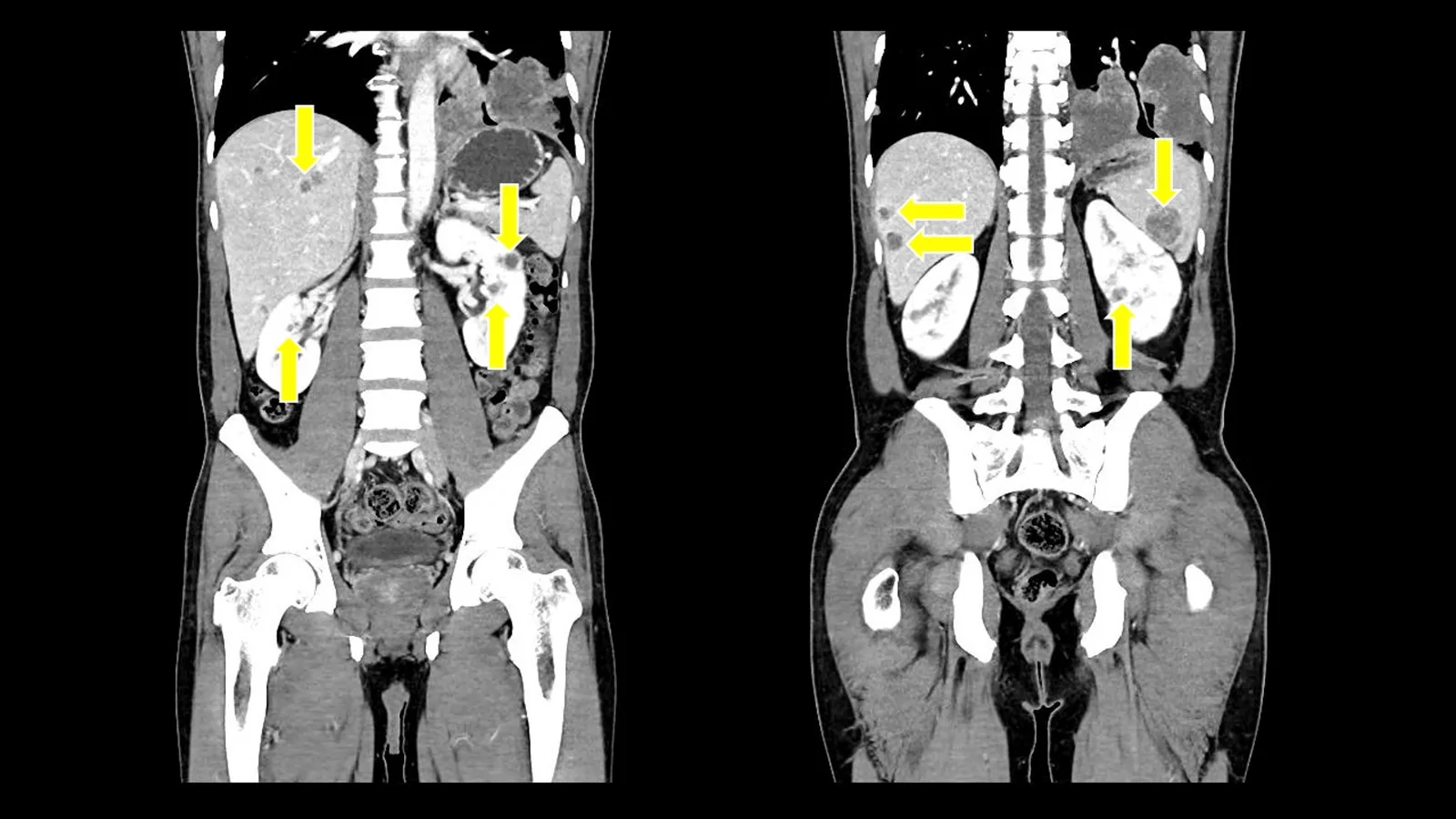
Contrast-enhanced CT of the abdomen for further evaluation Abdominal CT scan demonstrates scattered metastatic lesions (yellow arrows) involving the liver, spleen, and both kidneys, including a few focal hepatic lesions and a tiny splenic lesion CT: computed tomography

**Figure 6:**
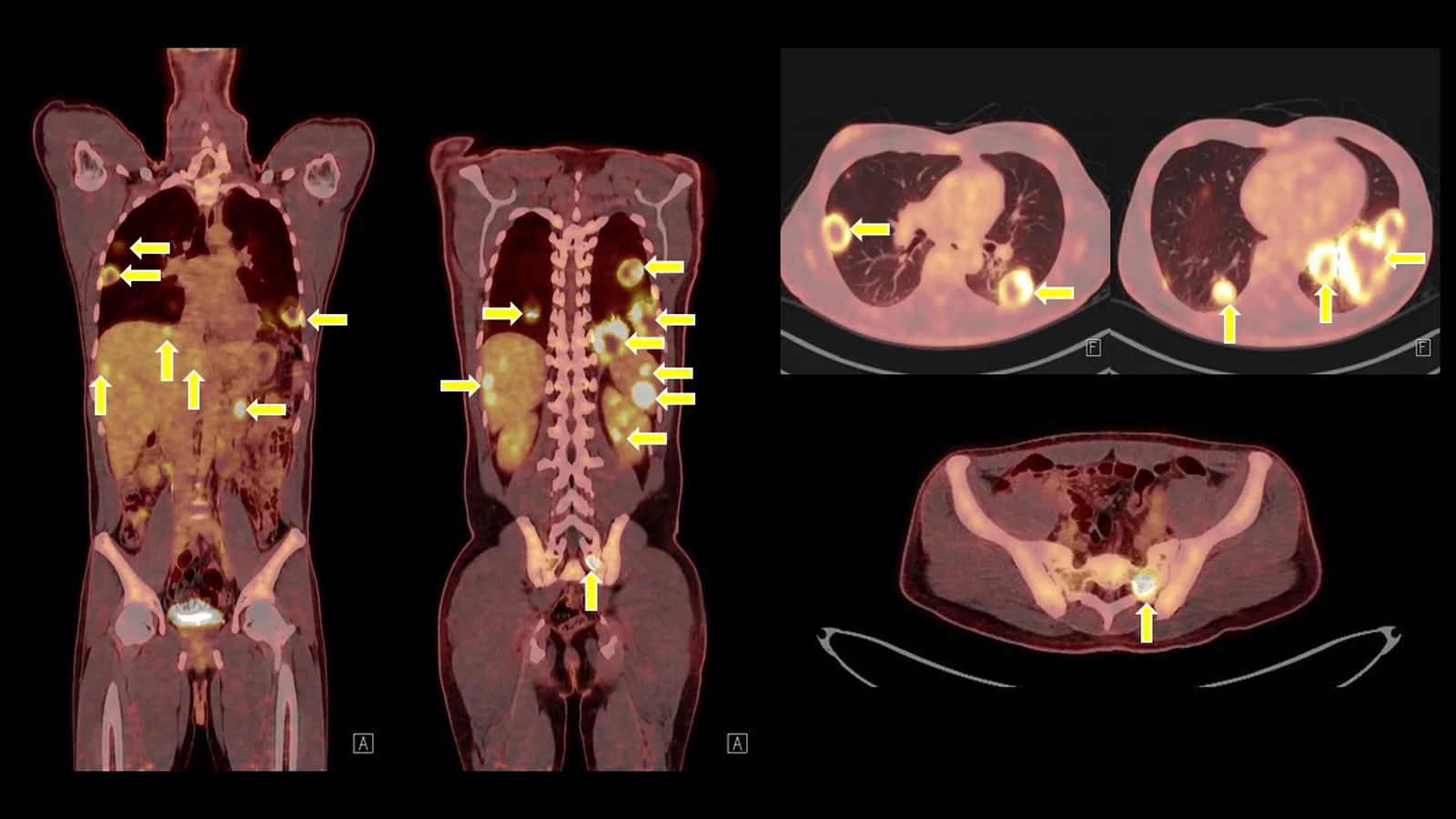
Whole-body FDG PET-CT demonstrating extensive systemic metastases 18F-FDG PET-CT images in coronal and axial views reveal multiple areas of intense hypermetabolism. These hypermetabolic lesions are distributed across the lungs, liver, spleen, bilateral kidneys, and the iliac bone, indicating widespread systemic metastatic disease (yellow arrows) FDG PET-CT: fluorodeoxyglucose positron emission tomography-computed tomography

**Figure 7:**
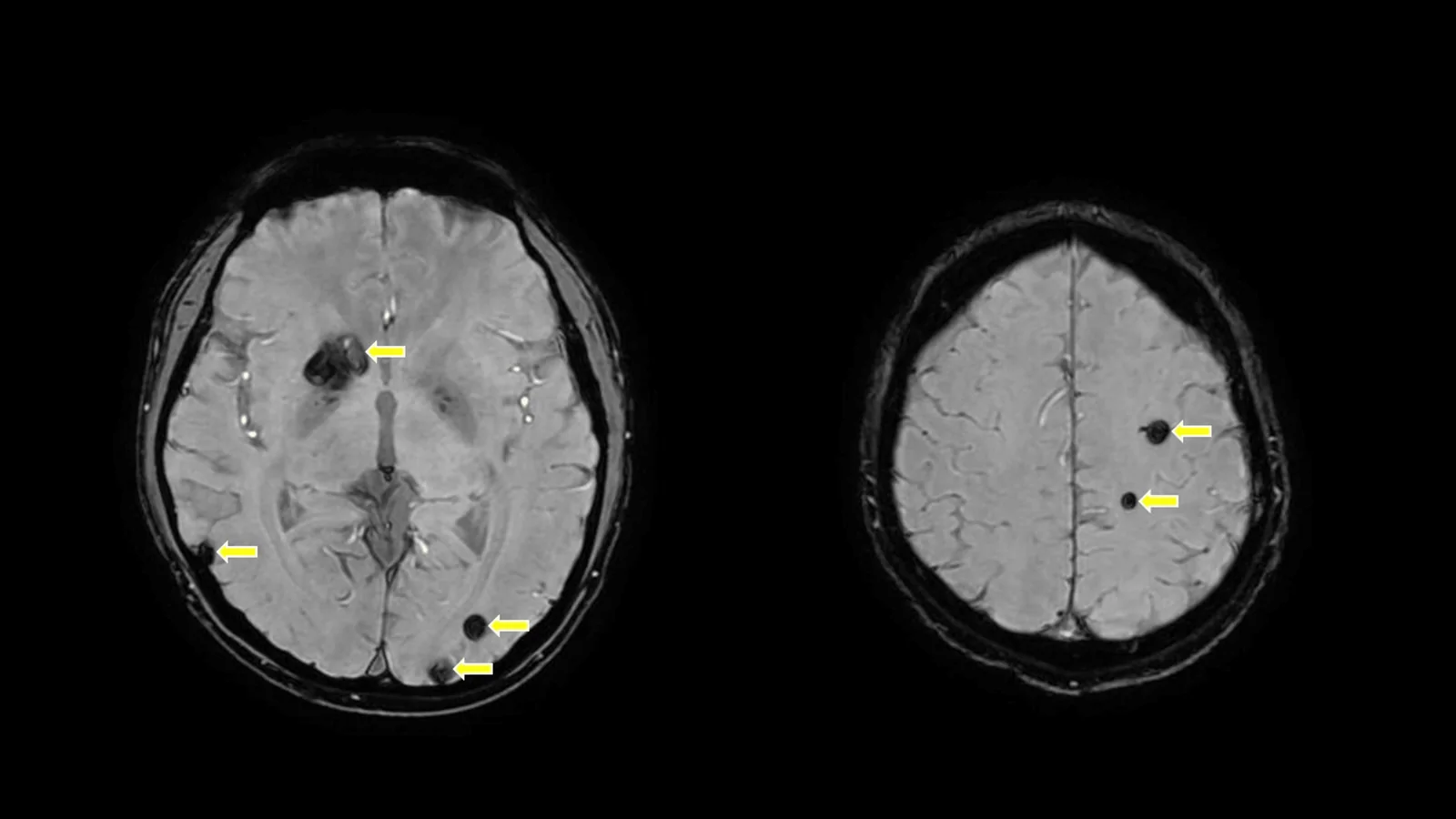
Brain SWI performed for the evaluation of motor weakness and headache SWI demonstrates multiple hemorrhagic metastatic lesions within the brain parenchyma (yellow arrows). In the setting of disseminated choriocarcinoma, these findings may reflect the characteristic vascular and hemorrhagic propensity of choriocarcinoma metastases SWI: susceptibility-weighted imaging

Subsequently, he presented with an abnormal chest radiograph, which demonstrated multiple increased opacities in both lung fields that were absent on imaging performed two months earlier (Figure 3). Multimodal imaging, including contrast-enhanced CT and PET/CT, revealed multiple systemic metastases involving the liver, spleen, pancreas, sacrum, and iliac bones (Figures 4, 5, 6). He was referred to hematology-oncology and initiated on VIP chemotherapy (etoposide, ifosfamide, cisplatin). During treatment, he developed acute right upper limb weakness. A follow-up contrast-enhanced brain MRI revealed multiple metastases in bilateral hemispheres with vasogenic edema and associated microbleeds on SWI (Figure 7). 

**Figure 8:**
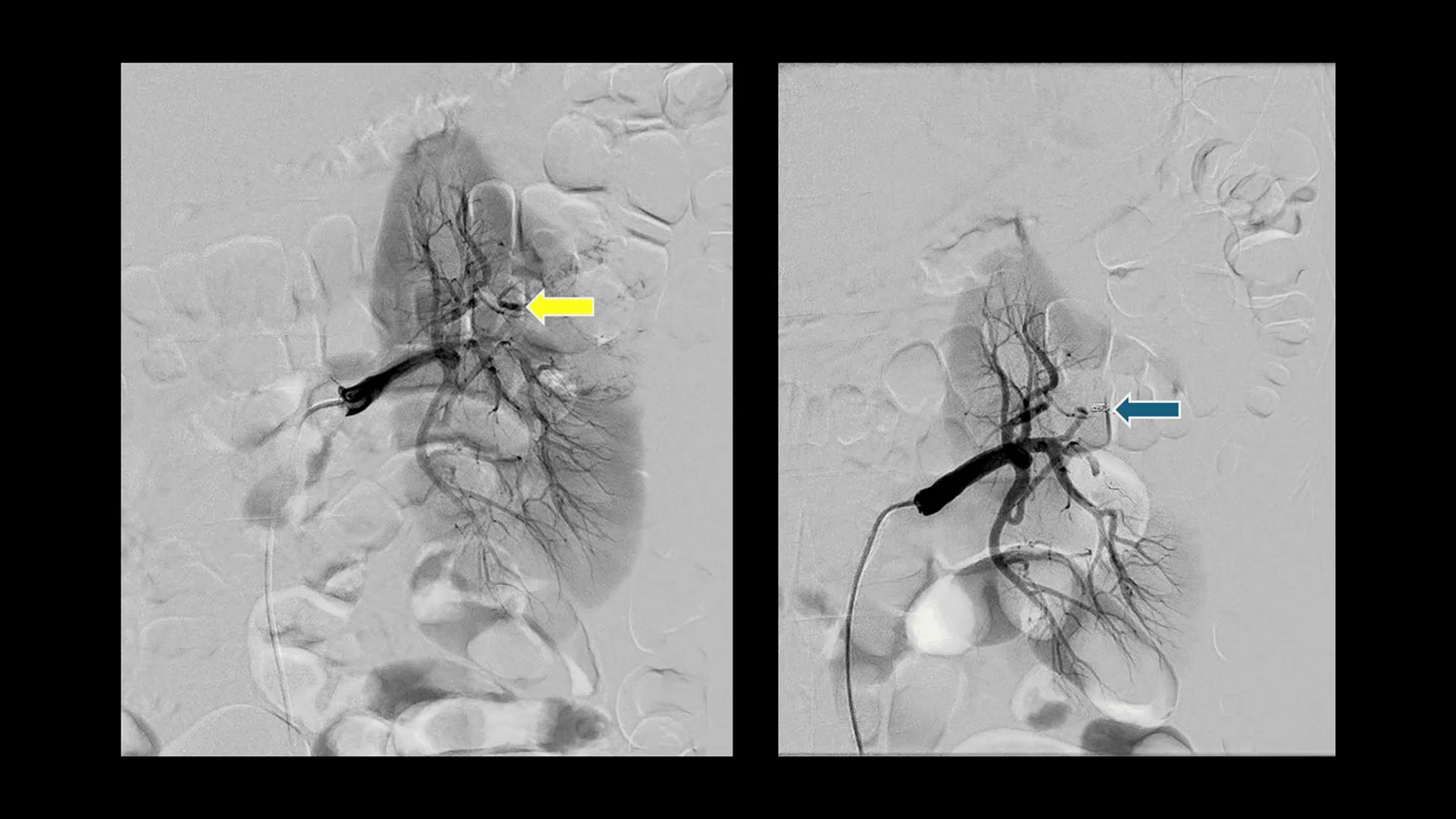
Renal angiogram for the evaluation and treatment of abdominal bleeding (A) Digital subtraction angiography demonstrates a pseudoaneurysm arising from a mid-pole branch of the renal artery (yellow arrow). (B) Post-embolization angiography demonstrates successful occlusion of the pseudoaneurysm (blue arrow)

**Figure 9:**
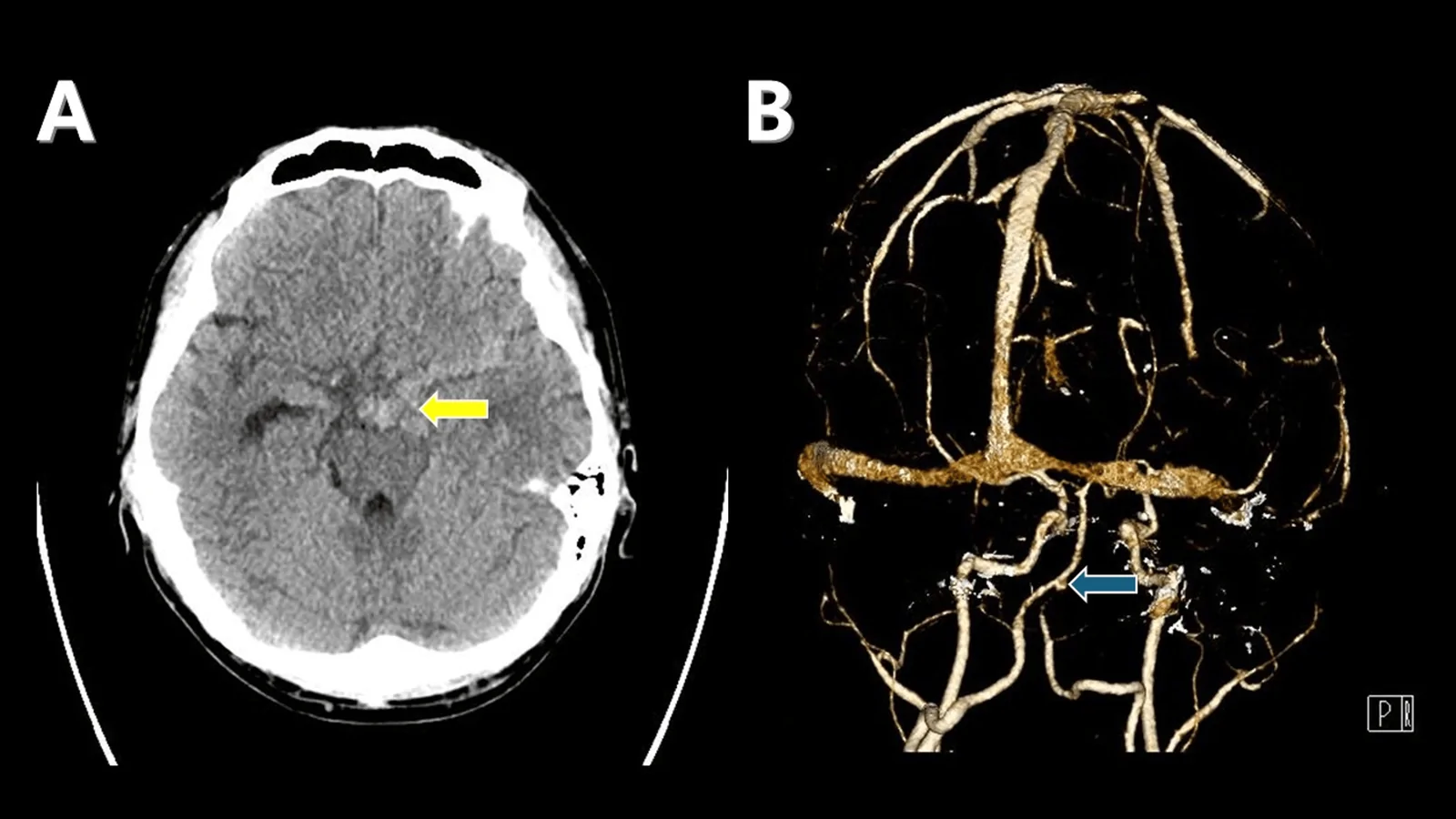
CTA showing a suspected cerebral aneurysm after a revisit to the emergency room for headache (A) A non-contrast brain CT scan performed upon presentation to the emergency room for headache reveals evidence of a subarachnoid hemorrhage (yellow arrow). (B) A 3D reconstructed CTA image shows a suspected aneurysm in an atypical location at the VB junction (blue arrow) CTA: computed tomography angiography; VB: vertebrobasilar

Whole-brain radiotherapy was administered at a total dose of 30 Gy in 10 fractions. Follow-up brain MRI demonstrated a reduction in the size of the nodular-enhancing metastatic lesions compared with the previous study. Subsequent contrast-enhanced brain MRIs revealed a continued decrease in the size of the metastatic lesions. Later that year, the patient presented with left flank pain. Abdominal CT demonstrated renal hemorrhage with an associated fluid collection. Renal artery embolization was performed using coils and Gelfoam, revealing tumor staining and a pseudoaneurysm in a branch of the middle pole (Figure 8) [4]. Shortly thereafter, the patient presented with neck stiffness and headache without fever. Contrast-enhanced brain MRI showed ventriculomegaly and ependymal enhancement, raising suspicion of meningitis. However, cerebrospinal fluid was grossly bloody, suggesting subarachnoid hemorrhage. Brain CT angiography was performed to rule out aneurysmal rupture and revealed a suspicious outpouching at the vertebrobasilar junction near the confluence of the bilateral vertebral arteries (Figure 9).

**Figure 10:**
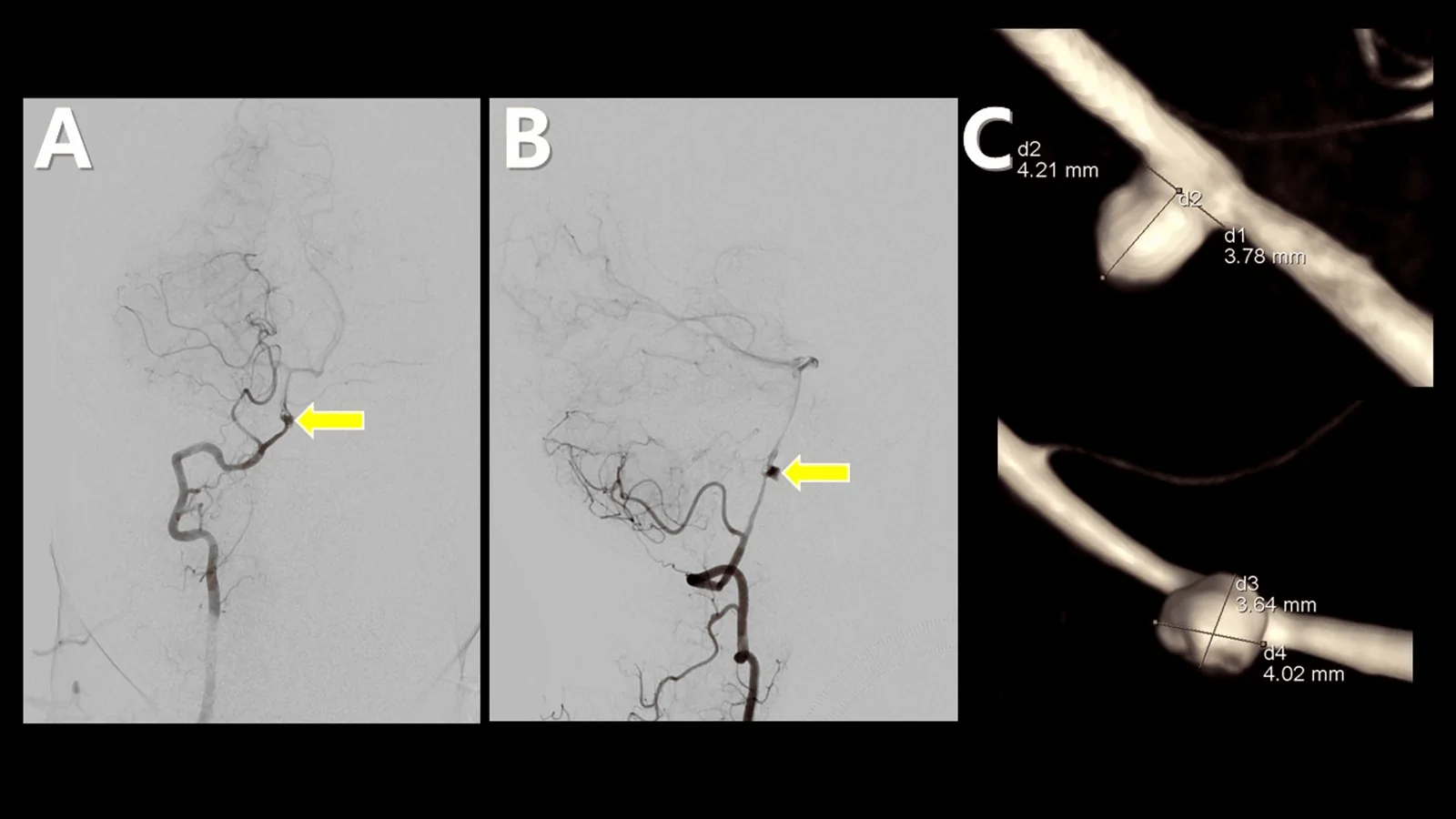
Digital subtraction angiography and 3D reconstruction images (A) Digital subtraction angiography in the frontal (anteroposterior) projection. (B) Digital subtraction angiography in the lateral projection. (C) 3D rotational angiographic reconstruction

Given the patient’s clinical history and imaging findings, tumor-related vascular rupture associated with leptomeningeal carcinomatosis was initially suspected rather than spontaneous SAH from an aneurysmal rupture. The patient experienced symptomatic relief from headache following a lumbar puncture and was admitted to the hematology-oncology department for further management. On hospital day 3, the onset of seizures prompted a follow-up MRI, which confirmed SAH with intraventricular hemorrhage. Endovascular coil embolization was planned following diagnostic digital subtraction angiography (DSA). DSA revealed a saccular aneurysm arising from the vertebrobasilar junction, an atypical location for such lesions (Figure 10). 

**Figure 11:**
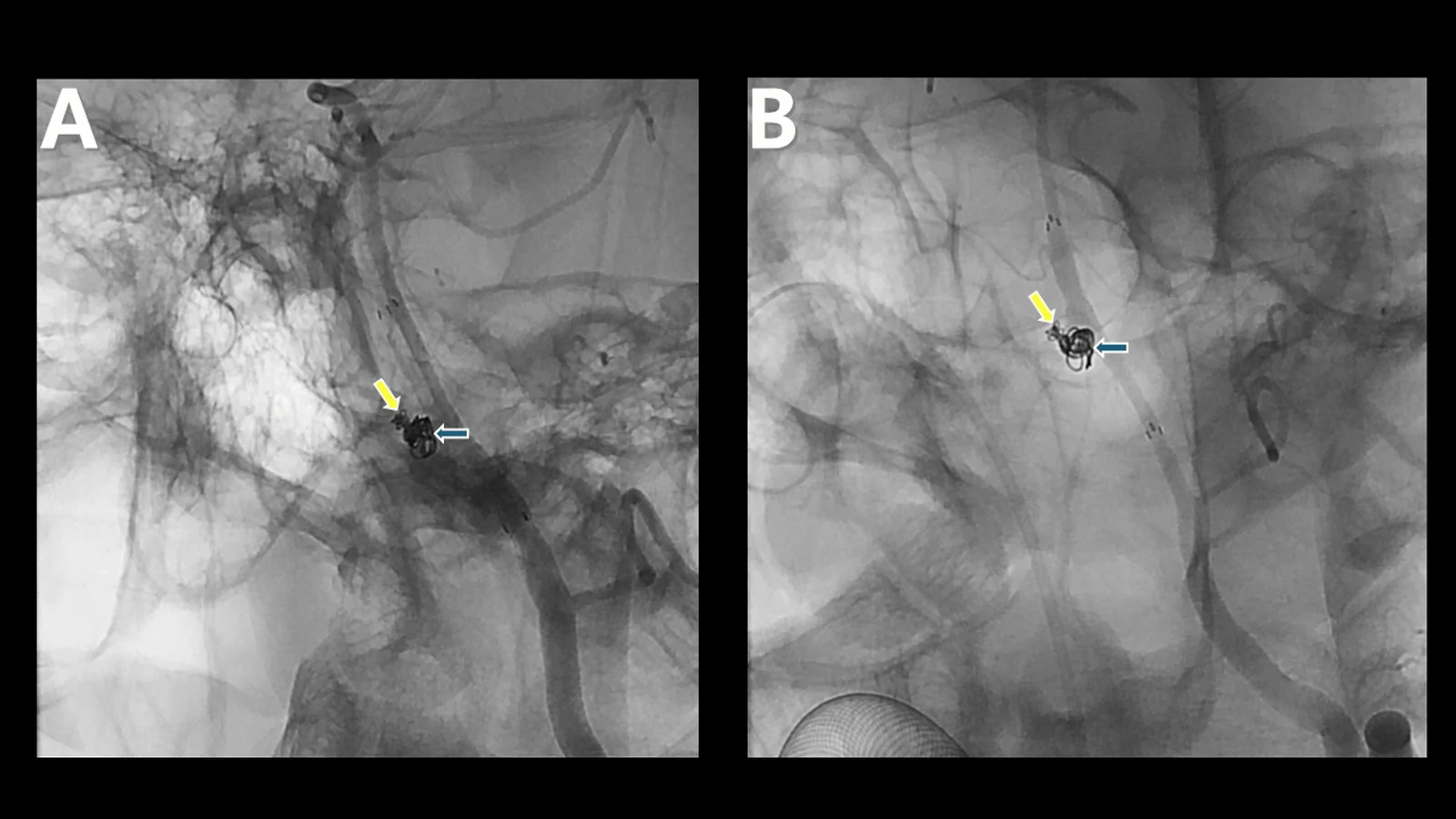
Comparison of angiograms from the initial and secondary coil embolization (A) An angiogram following the initial stent-assisted coil embolization shows a small filling coil resting upon the framing coil (yellow arrows). This is believed to be due to the extremely fragile intima, which provided insufficient support for the framing coil, preventing the filling coil from being positioned correctly. (B) Compared to the initial embolization image, a noticeable change in the coil shape is observed (blue arrows). This morphological alteration occurred only six days after the first procedure, representing a highly unusual case

Stent-assisted coil embolization was performed using Target 360 Ultra, Tetra, and Nanocoils supported by a Neuroform ATLAS stent (Stryker, Kalamazoo, MI). Post-procedural angiography showed no active contrast extravasation. An external ventricular drain (EVD) was placed to manage the increased intracranial pressure (ICP). The patient was sedated using remifentanil and propofol to maintain clinical stability. On postoperative day (POD) six, the patient developed acute pupillary dilation and loss of light reflex, accompanied by massive bloody drainage through the EVD. Emergency brain CT revealed significant rebleeding. Emergency coil embolization was immediately performed. The diagnostic angiogram revealed deformation of the coil mass (Figure 11).

Contrast opacification was significantly sluggish because of extremely high ICP. Transcellular catheterization through the existing stent struts was achieved, and the residual neck was packed using Target Nano coils. Unexpectedly, a coil migrated into the right distal P1 segment. A Neuroform ATLAS stent was required at the migration site to secure the coil. Post-angiogram and 3D DSA revealed no evidence of parental vessel occlusion. Severe vasospasm was suspected due to markedly sluggish contrast opacification, and diagnostic angiography of the bilateral internal carotid arteries was performed. Angiography revealed more severe vasospastic changes in the left MCA than in the right MCA. Consequently, 4 mg of intra-arterial nimodipine was administered via a microcatheter [5]. The control angiogram showed improvement in the vascular caliber of the left MCA. The femoral sheath was left in place for potential re-intervention. Despite these interventions, follow-up CT showed global cerebral edema with complete loss of gray-white matter differentiation, indicating irreversible brain injury [6].

## Discussion

Aneurysms at the VB junction are rare, largely due to the unique anatomical characteristics of this region [7,8]. The merging of high-velocity blood flow from each vertebral artery leads to expansion of the cross-sectional area and causes a rapid decrease in wall shear stress (WSS) at the junction apex [8]. Subsequently, the oscillatory shear index (OSI) tends to increase, with foci of high OSI directly coinciding with the low WSS regions [8]. The combination of decreased WSS, high OSI, and the formation of recirculation areas creates a hemodynamic environment that heavily favors the initiation of atherosclerotic changes [7,8]. Therefore, the VB junction predominantly exhibits atherosclerotic stenosis rather than aneurysmal changes. Through this hemodynamic mechanism, strong secondary flow and recirculation zones develop at the VB junction [7,8].

Consequently, tumor cells in the bloodstream may exhibit prolonged residence time within these recirculation zones. This region provides an optimal environment for tumor cells to adhere to the endothelium, primarily owing to the abnormally low WSS, which exerts minimal frictional force on the vessel wall [8]. Once attached to the intima, viable tumor cells are hypothesized to penetrate the endothelium and invade the vessel wall [3]. Trophoblastic choriocarcinoma, in particular, is widely recognized as angioinvasive, with these cells potentially capable of rapidly penetrating and destroying the vascular wall, including the internal elastic lamina [9,10].

**Figure 12:**
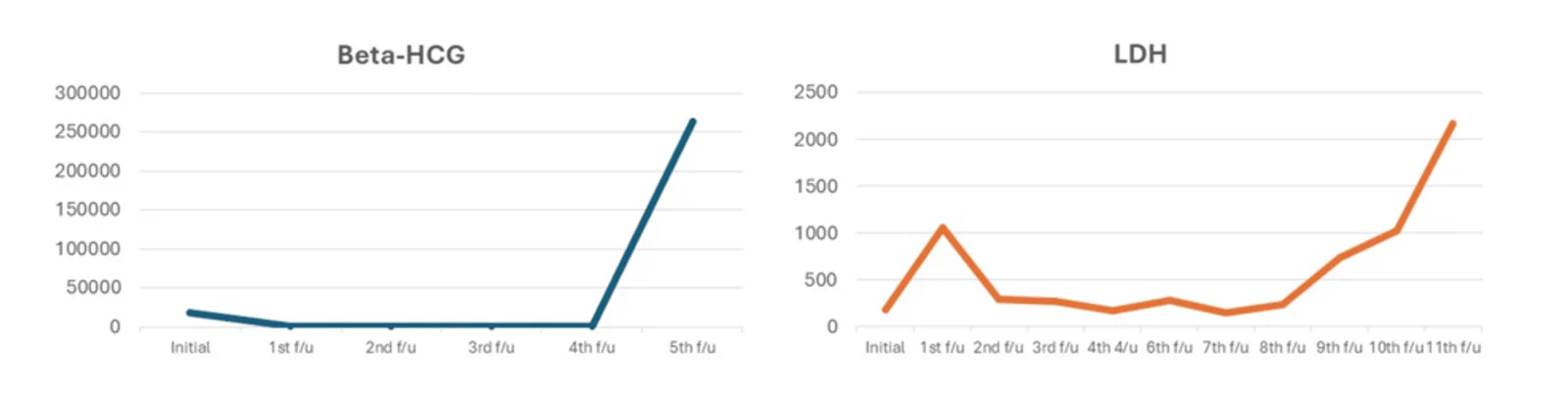
Trend graphs of beta-hCG and LDH levels The graphs illustrate the changes in (A) beta-hCG and (B) LDH levels over time. The levels were elevated at the time of initial diagnosis but stabilized following appropriate treatment. However, a rapid increase in both markers is observed just before the occurrence of the SAH. This sudden surge strongly suggests refractory recurrent choriocarcinoma hCG: human chorionic gonadotropin; LDH: lactate dehydrogenase; SAH: subarachnoid hemorrhage

This process is thought to result in structural weakening and predispose to aneurysm formation [3,9,10]. The history of renal artery pseudoaneurysm treated with coil embolization further supports the evidence of this oncogenic cerebral aneurysm. Given the highly angioinvasive nature of choriocarcinoma, this metastatic choriocarcinoma may have caused the destruction of vasculature across multiple organ systems [4]. Tumor markers, particularly LDH and β-hCG, demonstrated a marked increase, coinciding with the onset of spontaneous SAH and the need for stent-assisted coil embolization (Figure 12) [11-13].

**Figure 13:**
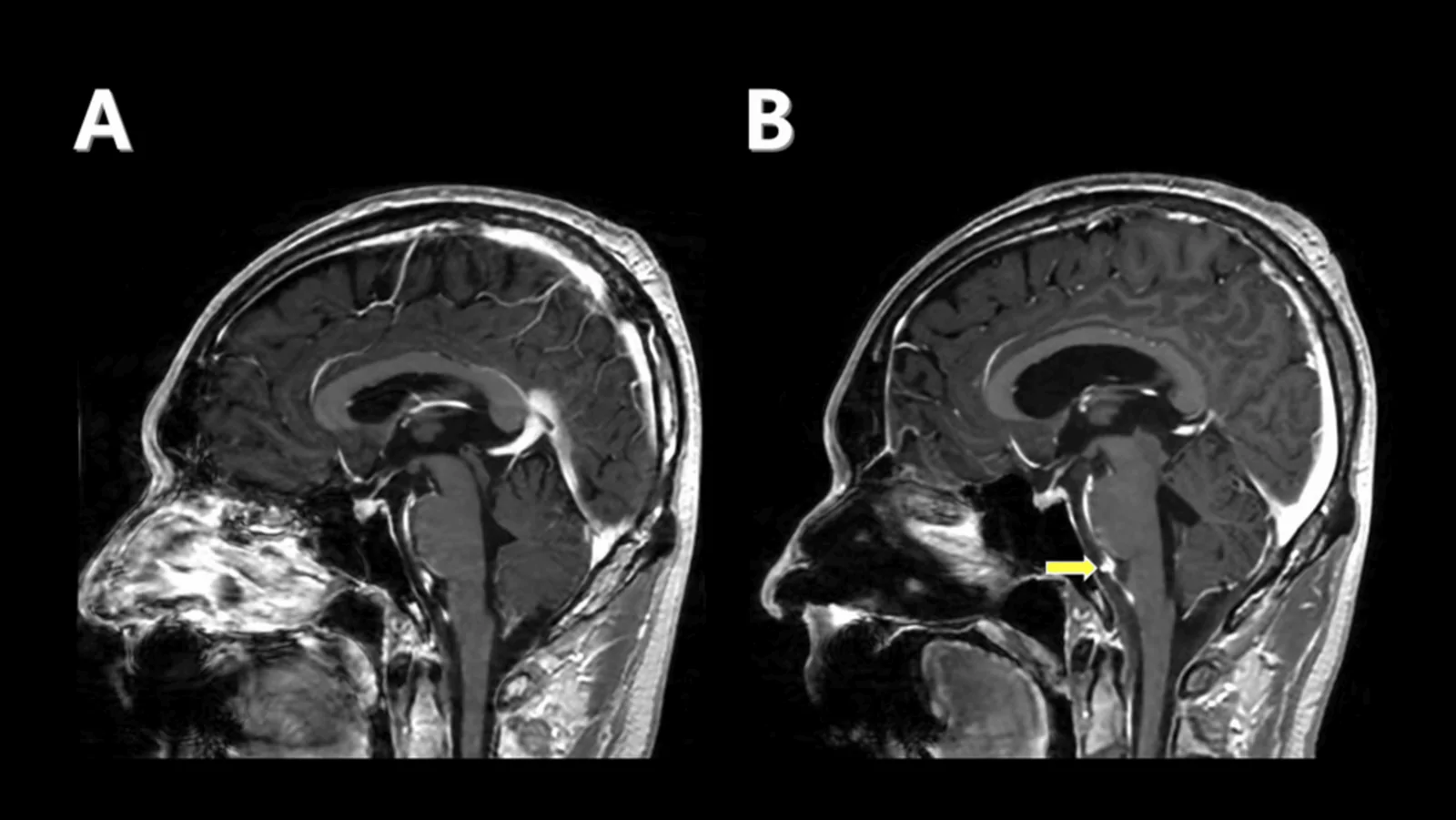
Comparison of follow-up contrast-enhanced brain MRI scans (A) A contrast-enhanced brain MRI from the year before the onset of SAH shows no outpouching lesion near the VB junction. (B) A follow-up MRI performed in the year of the SAH event reveals a suspected aneurysm near the VB junction (yellow arrow). The appearance of this lesion closely coincides with the timing of the rapid surge in beta-hCG levels MRI: magnetic resonance imaging; SAH: subarachnoid hemorrhage; VB: vertebrobasilar; hCG: human chorionic gonadotropin

Despite ongoing chemotherapy, elevated β-hCG suggests a refractory disease course and irreversibly damaged cerebral vasculature [12-14]. Follow-up MRI after brain metastasis and whole-brain radiotherapy demonstrated stable vasculature at the VB junction until the year before the onset of SAH. However, an MRI performed just before the onset of SAH showed an abnormal outpouching from the VB junction, consistent with the progressive increase of β-hCG (Figure 13) [13-15]. Pre-coiling DSA demonstrated a typical saccular aneurysm at the VB junction, for which stent-assisted coil embolization was performed.

**Figure 14:**
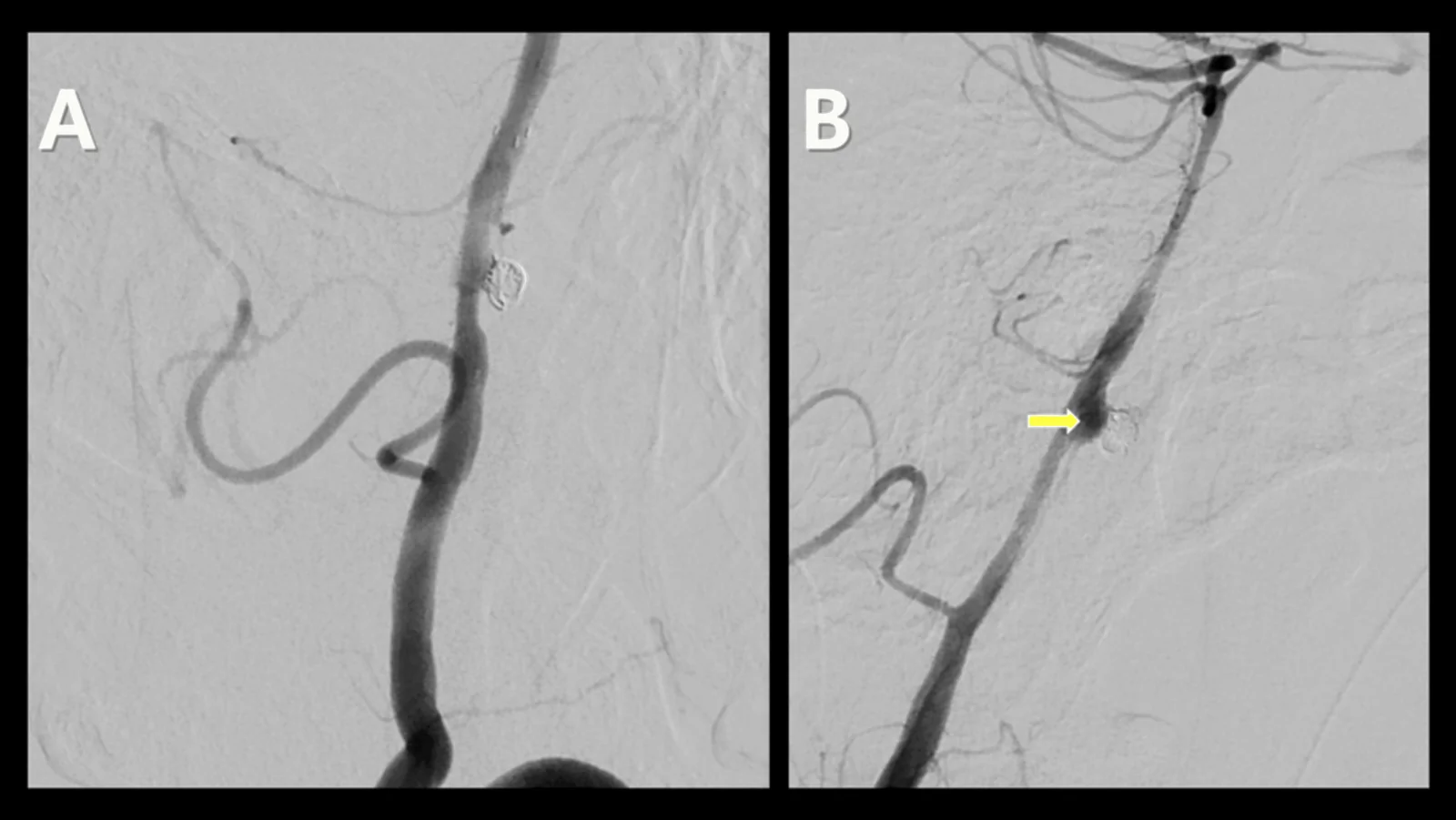
Comparison of angiograms between the first and second coil embolizations (A) During the initial coil embolization, a small residual neck was observed. (B) An angiogram performed during the second coil embolization revealed a growing neck (yellow arrow) compared to the first procedure. This finding suggests that the angioinvasive choriocarcinoma continuously destroyed the vascular integrity, despite the initial coil embolization

Typically, smaller coils are expected to be packed within the framing coil; however, the final angiogram demonstrated an accessory small coil mass positioned above the framing coil, suggesting reduced structural integrity of the vascular intima (Figure 12) [9,10,13,15]. This finding indicates that vessel wall fragility may have limited stable coil integration [8-10,15]. Similar features were also observed during repeat embolization performed for re-bleeding. DSA showed deformation of the coil mass, and the angiogram revealed a growing neck (Figure 14). Although the bleeding source was not confirmed due to increased ICP and unidentified active contrast extravasation during the second procedure, potential sites included the growing neck of the pseudoaneurysm or the whole cerebral vasculature.

## Conclusions

Given these findings, neurointerventionists may consider atypical sites of OCAs, including the vertebrobasilar (VB) junction, particularly in the setting of metastatic choriocarcinoma. While proactive cerebrovascular screening may be considered in patients with rising β-hCG levels, its general clinical utility requires further validation. As demonstrated in this case, when metastatic choriocarcinoma exhibits aggressive behavior, endovascular treatment may pose technical challenges related to vascular structural integrity due to increased vascular fragility. Furthermore, because recurrent hemorrhage may arise from diffuse vascular vulnerability despite successful initial treatment, a multidisciplinary approach involving hematology-oncology specialists is essential. In such a complex scenario, alternative strategies like stent-overlapping techniques may be considered as potential options rather than definitive recommendations.
